# Adenosinergic Depression of Glutamatergic Transmission in the Entorhinal Cortex of Juvenile Rats via Reduction of Glutamate Release Probability and the Number of Releasable Vesicles

**DOI:** 10.1371/journal.pone.0062185

**Published:** 2013-04-16

**Authors:** Shouping Wang, Lalitha Kurada, Nicholas I. Cilz, Xiaotong Chen, Zhaoyang Xiao, Hailong Dong, Saobo Lei

**Affiliations:** 1 Department of Pharmacology, Physiology and Therapeutics, School of Medicine and Health Sciences, University of North Dakota, Grand Forks, North Dakota, United States of America; 2 Department of Anesthesiology, Sun Yat-sen Memorial Hospital, Sun Yat-sen University, Guangzhou, P.R. China; 3 Department of Emergency Medicine, Sun Yat-sen Memorial hospital, Sun Yat-sen University, Guangzhou, P.R. China; 4 Department of Anesthesiology, Xijing Hospital, Fourth Military Medical University, Xi'an, P.R. China; University of North Dakota, United States of America

## Abstract

Adenosine is an inhibitory neuromodulator that exerts antiepileptic effects in the brain and the entorhinal cortex (EC) is an essential structure involved in temporal lobe epilepsy. Whereas microinjection of adenosine into the EC has been shown to exert powerful antiepileptic effects, the underlying cellular and molecular mechanisms in the EC have not been determined yet. We tested the hypothesis that adenosine-mediated modulation of synaptic transmission contributes to its antiepileptic effects in the EC. Our results demonstrate that adenosine reversibly inhibited glutamatergic transmission via activation of adenosine A_1_ receptors without effects on GABAergic transmission in layer III pyramidal neurons in the EC. Adenosine-induced depression of glutamatergic transmission was mediated by inhibiting presynaptic glutamate release probability and decreasing the number of readily releasable vesicles. Bath application of adenosine also reduced the frequency of the miniature EPSCs recorded in the presence of TTX suggesting that adenosine may interact with the exocytosis processes downstream of Ca^2+^ influx. Both Gα_i/o_ proteins and the protein kinase A pathway were required for adenosine-induced depression of glutamatergic transmission. We further showed that bath application of picrotoxin to the EC slices induced stable epileptiform activity and bath application of adenosine dose-dependently inhibited the epileptiform activity in this seizure model. Adenosine-mediated depression of epileptiform activity was mediated by activation of adenosine A_1_ receptors and required the functions of Gα_i/o_ proteins and protein kinase A pathway. Our results suggest that the depression of glutamatergic transmission induced by adenosine contributes to its antiepileptic effects in the EC.

## Introduction

The entorhinal cortex (EC) mediates the majority of connections between the hippocampus and other cortical areas [1], [2]. Inputs from the olfactory structures, parasubiculum, presubiculum, perirhinal cortex, claustrum, amygdala and neurons in the deep layers of the EC (layers V–VI) [1], [3], [4] converge onto the superficial layers (layer II/III) of the EC whereas the axons of principal neurons in layer II of the EC form the major component of perforant path that innervates the dentate gyrus and CA3 [5] and the axons of layer III pyramidal neurons form the temporoammonic pathway that synapses onto the distal dendrites of pyramidal neurons in CA1 and subiculum [2], [5], [6]. Furthermore, neurons in the deep layers of the EC relay a large portion of hippocampal output information back to the superficial layers [7], [8], [9], [10] and to other cortical areas [1]. The functions of the EC are involved in emotional control [11], consolidation and recall of memories [12], [13], Alzheimer's disease [14], [15], schizophrenia [16], [17] and temporal lobe epilepsy [18], [19].

As an inhibitory neuromodulator in the brain [20], [21], adenosine modulates a variety of physiological functions including sleep [20], [22], nociception [23], cerebral blood flow [24] and respiration [25] as well as many neurological disorders such as epilepsy [26], Parkinson disease [27], [28] and Huntington disease [29]. Adenosine interacts with 4 subtypes of G protein-coupled adenosine receptors (ARs) that include A_1_, A_2A_, A_2B_ and A_3_ [20], [21], [30], [31]. The A_1_ ARs are coupled to Gα_i_ proteins leading to inhibition of adenylyl cyclase (AC)-cAMP-protein kinase A (PKA) pathway whereas the other three ARs are coupled to G_s_ proteins resulting in activation of AC-cAMP-PKA pathway [21]. Furthermore, activation of A_1_ ARs activates phospholipase A_2_ and phospholipase D whereas A_2B_ and A_3_ receptors increase the function of phospholipase C [21]. The biological functions of adenosine are likely to be mediated by these receptors.

Adenosine-mediated antiepileptic effects have been observed in the EC. Activation of A_1_ ARs prevents Mg^2+^-free-induced seizure-like events recorded from *in vitro* EC slices [32]. Microinjection of selective A_1_ AR agonist into the EC of the intact animals inhibits epileptic activity [33], [34]. However, the cellular and molecular mechanisms underlying adenosine-induced antiepileptic effects in the EC have not been determined yet. Whereas glutamate is the major excitatory neurotransmitter in the EC, the roles of adenosine on glutamatergic transmission in the EC have not been determined. In the present study, we examined the effects of adenosine on glutamatergic transmission and epileptiform activity in the EC. We focused on layer III pyramidal neurons because selective loss of layer III pyramidal neurons in the EC has been observed in epileptic animals [35], [36] highlighting the importance of these neurons in epilepsy. Our results demonstrated that adenosine exerts remarkable inhibition of glutamate release and epileptiform activity by A_1_ AR-mediated down-regulation of AC-cAMP-PKA pathway resulting in decreases of presynaptic release probability and the number of readily releasable vesicles. Our results provide a cellular and molecular mechanism that helps explain adenosine-induced antiepileptic effects in the EC.

## Materials and Methods

### Slice preparation

Horizontal brain slices (400 µm) including the EC, subiculum and hippocampus were cut using a vibrating blade microtome (VT1000S; Leica, Wetzlar, Germany) from 12- to 18-day-old Sprague Dawley rats as described previously [37], [38], [39], [40]. Briefly, after being deeply anesthetized with isoflurane, rats were decapitated and their brains were dissected out in ice-cold saline solution that contained (in mM) 130 NaCl, 24 NaHCO_3_, 3.5 KCl, 1.25 NaH_2_PO_4_, 0.5 CaCl_2_, 5.0 MgCl_2_, and 10 glucose, saturated with 95% O_2_ and 5% CO_2_ (pH 7.4). Slices were initially incubated in the above solution at 35°C for 40 min for recovery and then kept at room temperature (∼24°C) until use. All animal procedures conformed to the guidelines of the University of North Dakota Animal Care and Use Committee. This specific study was approved by the University of North Dakota Animal Care and Use Committee.

### Recordings of synaptic currents

**Figure 1:**
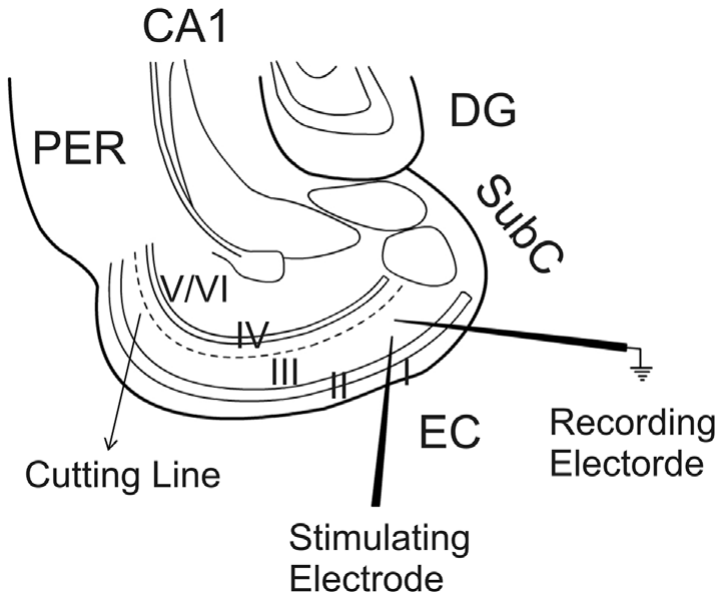
Diagram showing the location and different layers of the EC. Dotted line shows the location of cutting. Recordings were conducted form layer III pyramidal neurons with a stimulation electrode placed in ∼200 µm from the recorded neuron in layer III. DG, dentate gyrus; Subc, subiculum; PER, perirhinal; EC, entorhinal cortex.

Whole-cell patch-clamp recordings using an Axopatch 200B or two Multiclamp 700B amplifiers in voltage-clamp mode from *in vitro* entorhinal slices were used for experiments. Layer III pyramidal neurons in the medial EC were visually identified with infrared video microscopy and differential interference contrast optics [41], [42], [43], [44]. Recording electrodes were filled with the solution containing (in mM) 100 Cs-gluconate, 0.6 EGTA, 5 MgCl_2_, 8 NaCl, 2 ATP_2_Na, 0.3 GTPNa, 40 HEPES and 1 QX-314 (pH 7.3). The extracellular solution (ACSF) comprised (in mM) 130 NaCl, 24 NaHCO_3_, 3.5 KCl, 1.25 NaH_2_PO_4_, 2.5 CaCl_2_, 1.5 MgCl_2_ and 10 glucose (saturated with 95% O_2_ and 5% CO_2_, pH 7.4) unless stated otherwise. Bicuculline (10 µM) was included in the extracellular solution to block GABA_A_ receptors. To prevent the propagation of epileptic activity in the presence of bicuculline, a cut was made between layer III and layer V with a microknife (Catalog number: RS-6242, Roboz Surgical Instrument Company, Gaithersburg, Maryland) under a microscope before the slices were transferred to the recording chamber [45] (Fig. 1). The holding potential was at −65 mV unless stated otherwise. AMPA receptor-mediated EPSCs were evoked by placing a stimulation electrode in layer III about 200 µm from the recorded neuron (Fig. 1). For the isolation of NMDA EPSCs, the extracellular solution contained DNQX (10 µM) to block AMPA/kainate receptors and bicuculline (10 µM) to block GABA_A_ receptors and the holding potential was at +40 mV. Series resistance was rigorously monitored by the delivery of 5 mV voltage steps after each evoked current. Experiments were discontinued if the series resistance changed by >15%. Miniature AMPA EPSCs (mEPSCs) were recorded from layer III pyramidal cells of the EC in the presence of TTX (1 µM). Data were filtered at 2 kHz, digitized at 10 kHz, acquired on-line and analyzed after-line using pCLAMP 9 software (Molecular Devices, Sunnyvale, CA). The recorded mEPSCs were analyzed afterwards using Mini Analysis 6.0.1 (Synaptosoft Inc., Decatur, GA, USA). To avoid potential desensitization, only one cell was recorded from each slice for each experiment.

### Recordings of the spontaneous seizure activity

Spontaneous seizure activity was induced from *in vitro* slices by including GABA_A_ receptor blocker, picrotoxin (100 µM), in the preceding ACSF except that the final concentration of KCl was increased to 5 mM to increase the frequency of spontaneous seizure activity. An electrode containing this ACSF without picrotoxin was placed in layer III of the EC to record seizure activity. After stable spontaneous seizure activity occurred, adenosine (100 µM) was applied in the bath. The seizure events were initially recorded by Clampex 9 and subsequently analyzed by Mini Analysis 6.0.1.

### Data analysis

Data are presented as the means ± S.E.M. Concentration-response curve of adenosine was fit by Hill equation: *I* = *I*_max_×{1/[1+(EC_50_/[ligand])*^n^*]}, where *I*_max_ is the maximum response, EC_50_ is the concentration of ligand producing a half-maximal response, and *n* is the Hill coefficient. Coefficient of variation (CV) was calculated by the equation, CV = SD/X, where SD is the standard deviation and X is the mean of 15 consecutive AMPA EPSCs. The paired-pulse ratio (PPR) was calculated as the mean P2/mean P1, where P1 was the amplitude of first evoked current and P2 was the amplitude of the second synaptic current, measured after subtraction of the remaining P1 ‘tail’ current [46], [47]. For mEPSC cumulative probability plots, events recorded for 4 min before adenosine application and 4 min after the maximal effect of adenosine were selected. Same bin size (25 ms for frequency and 1 pA for amplitude) was used to analyze data from control and adenosine treatment. Kolmogorov-Smirnoff (KS) test was used to assess the significance of the cumulative probability plots. Student's paired or unpaired *t* test or analysis of variance (ANOVA) was used for statistical analysis as appropriate; P values are reported throughout the text and significance was set as P<0.05. N numbers in the text represent the number of cells examined unless stated otherwise. To reduce variation from individual animals, each experiment was conducted from slices cut from at least 3 rats.

### Chemicals

*N*-Cyclopentyladenosine (NCPA), 8-cyclopentyl-1,3-dipropylxanthine (DPCPX), 1-butyl-8-(hexahydro-2,5-methanopentalen-3a(1*H*)-yl)-3,7-dihydro-3-(3-hydroxypropyl)-1*H*-purine-2,6-dione (PSB36), 2-(2-Furanyl)-7-[3-(4-methoxyphenyl)propyl]-7*H*-pyrazolo[4,3-*e*][1], [2], [4]triazolo[1,5-*c*]pyrimidin-5-amine (SCH442416), 8-[4-[4-(4-Chlorophenzyl)piperazide-1-sulfonyl)phenyl]]-1-propylxanthine (PSB603), *N*-[9-Chloro-2-(2-furanyl)[1], [2], [4]-triazolo[1,5-*c*]quinazolin-5-yl]benzene acetamide (MRS1220), 6,7-dinitroquinoxaline-2,3-dione (DNQX), *dl*-2-amino-5-phosphonopentanoic acid (*dl*-AVP), pertussis toxin, KT5720 and MDL-12,330A were purchased from Tocris Cookson Inc. (Ellisville, MO). GDP-β-S and Rp-cAMPS were purchased from Enzo Life Sciences International, Inc. (Plymouth Meeting, PA). Other chemicals were products of Sigma-Aldrich (St. Lois, MO).

## Results

### Adenosine depresses glutamatergic but not GABAergic transmission onto layer III pyramidal neurons in the EC via activation of A_1_ ARs

**Figure 2:**
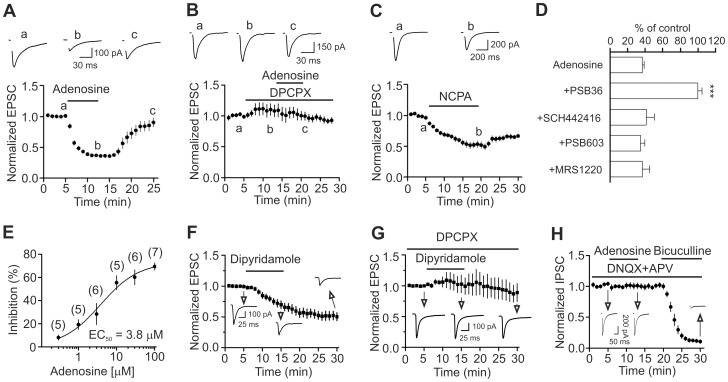
Adenosine decreases the amplitude of evoked AMPA EPSCs via activation of A_1_ ARs without altering that of the evoked IPSCs recorded from layer III pyramidal neurons in the medial EC. A, Bath application of adenosine (100 µM) reversibly inhibited the evoked AMPA EPSCs (n = 15, p<0.001 vs. baseline, paired t-test). Upper panel shows the average of 10 EPSCs recorded at different time points in the figure. Stimulation artifacts were blanked for clarity (same for the rest of the figures). **B,** Bath application of the A_1_ AR antagonist, DPCPX (1 µM) completely blocked adenosine-induced depression of AMPA EPSCs (n = 5, p = 0.8 vs. baseline, paired t-test). **C,** Bath application of the A_1_ AR agonist, NCPA (2 µM), inhibited AMPA EPSCs (n = 10, p<0.001 vs. baseline, paired t-test). **D,** Application of the antagonists for receptors other than A_1_ ARs failed to block adenosine-induced depression of AMPA EPSCs (One-way ANOVA followed by Dunnett test, *** p<0.001 vs. adenosine alone). **E,** Concentration-response curve of adenosine. The numbers in the parentheses are the numbers of cells used for each concentration. **F,** Bath application of dipyridamole (1 µM), an adenosine transporter inhibitor, significantly reduced the evoked EPSCs (n = 10, p<0.001, paired t-test) suggesting that endogenously released adenosine decreases AMPA EPSCs. **G,** Prior application of DPCPX, an A_1_ AR blocker, blocked dipyridamole-induced depression of AMPA EPSCs (n = 5, p = 0.85 vs. baseline, paired t-test) suggesting that the inhibitory effect of dipyridamole is mediated via activation of A_1_ ARs. **H,** Bath application of adenosine (100 µM) had no effects on the evoked IPSCs recorded at −70 mV from layer III pyramidal neurons (n = 7, p = 0.84 vs. baseline, paired t-test). The extracellular solution contained DNQX (20 µM) and *dl*-APV (100 µM). At the end of the experiments, application of bicuculline (20 µM) completely blocked IPSCs indicating that the recorded IPSCs were mediated by activation of GABA_A_ receptors.

We initially examined the effects of adenosine on synaptic transmission onto layer III pyramidal neurons in the medial EC. We recorded AMPA EPSCs from layer III pyramidal neurons by placing the stimulation electrode ∼200 µm from the recorded neurons in layer III (Fig. 1). Bath application of adenosine (100 µM) for 7 min induced remarkable depression of the amplitudes of evoked AMPA EPSCs (36±2% of control, n = 15, p<0.001, Fig. 2A). Adenosine-mediated depression was reversible. The amplitude of AMPA EPSCs returned to 90±8% of control after wash in adenosine-free extracellular solution for 13 min (n = 15, p = 0.22 vs. baseline). We then tested the involvement of ARs in adenosine-induced depression of AMPA EPSCs. Adenosine interacts with four different types of ARs: A_1_, A_2A_, A_2B_ and A_3_. Application of DPCPX (1 µM), a selective A_1_ AR blocker, did not alter significantly AMPA EPSC amplitude (110±9% of control, n = 5, p = 0.35, Fig. 2B) but completely blocked adenosine-induced depression of AMPA EPSCs (102±9% of control, n = 5, p = 0.8, Fig. 2B) suggesting the involvement of A_1_ ARs. Similarly, application of PSB36 (1 µM), another selective A_1_ AR antagonist, did not change significantly AMPA EPSCs (109±4% of control, n = 5, p = 0.07) but completely blocked adenosine-induced inhibition of AMPA EPSCs (100±5% of control, n = 5, p = 0.96, Fig. 2D). In line with these results, bath application of the selective A_1_ AR agonist NCPA (2 µM) suppressed AMPA EPSCs to 53±5% of control (n = 10, p<0.001, Fig. 2C). However, adenosine-mediated depression of AMPA EPSCs were insignificantly altered in the presence of SCH442416 (1 µM), a selective A_2A_ AR antagonist (41±9% of control, n = 6, p = 0.44 vs. adenosine alone, Fig. 2D), or PSB603 (1 µM), a selective A_2B_ AR antagonist (35±5% of control, n = 6, p = 0.59 vs. adenosine alone, Fig. 2D), or MRS1220 (10 µM), a selective A_3_ AR antagonist (37±8% of control, n = 6, p = 0.99 vs. adenosine alone, Fig. 2D). These results unanimously indicate that adenosine-induced depression of AMPA EPSCs is mediated via activation of A_1_ ARs in the EC. The EC_50_ value of adenosine was measured to be 3.8 µM (Fig. 2E).

We then probed whether endogenously released adenosine modulates AMPA EPSCs. Bath application of dipyridamole (1 µM), an adenosine transporter inhibitor, significantly decreased AMPA EPSCs (70±6% of control, n = 10, p<0.001, Fig. 2F). AMPA EPSCs were persistently inhibited after application of dipyridamole for 10 min suggesting a continual inhibition of adenosine transporter. The inhibitory effect of dipyridamole was mediated via activation of A_1_ ARs because prior application of the selective A_1_ AR blocker, DPCPX (1 µM), completely blocked dipyridamole-induced depression of AMPA EPSCs (103±15% of control, n = 5, p = 0.85, Fig. 2G). These data together suggest that endogenous released adenosine inhibits AMPA EPSCs via activation of A_1_ ARs in the EC.

We also tested whether adenosine modulates GABAergic transmission in the EC. The extracellular solution contained *dl*-APV (50 µM) and DNQX (10 µM) to block glutamatergic responses. Under this condition, the reversal potential for the IPSCs was ∼ −43 mV in our recording conditions [45]. We therefore held the cells at −70 mV and recorded the evoked IPSCs by placing the stimulation electrode ∼200 µm from the recorded cells in layer III. Under these circumstances, bath application of adenosine (100 µM) did not significantly change the evoked IPSCs (101±5% of control, n = 7, p = 0.84, Fig. 2H) and subsequent application of bicuculline (10 µM) at the end of experiments blocked the evoked IPSCs demonstrating that adenosine does not modulate GABAergic transmission in the EC.

### Adenosine depresses AMPA EPSCs via inhibition of presynaptic glutamate release

**Figure 3:**
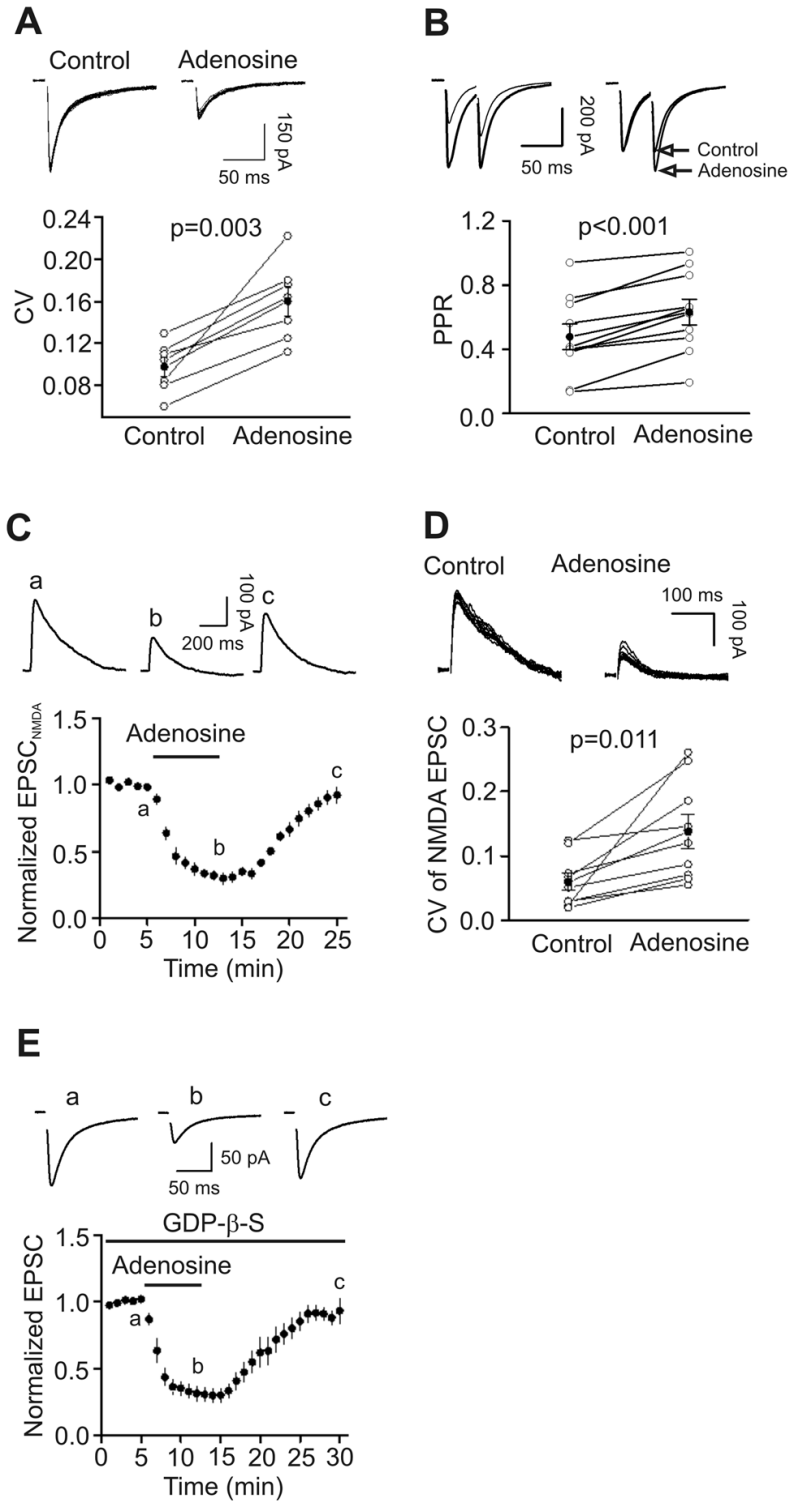
Adenosine inhibits AMPA EPSCs by decreasing presynaptic glutamate release. A, Application of adenosine increased the CV of AMPA EPSCs (n = 7, p = 0.003, paired t-test). Upper panel shows 15 consecutive AMPA EPSCs recorded before and during the application of adenosine. Lower panel shows the calculated CVs from 7 cells (*open circles*) and their averages (*solid circles*). **B,** Adenosine increased PPR (n = 10, p<0.001, paired t-test). *Upper left*, AMPA EPSCs evoked by two stimulations at an interval of 50 ms before and during the application of adenosine. *Upper right*, EPSCs recorded before and during the application of adenosine were scaled to the first EPSC. Note that the second EPSC after the application of adenosine is larger than control. *Bottom*, PPRs recorded from 7 cells (*open circles*) and their averages (*solid circles*). **C,** Application of adenosine inhibited NMDA EPSCs (n = 9, p<0.001 vs. baseline, paired t-test). Upper panel shows the averaged NMDA EPSC of 5 EPSCs at different time points in the figure. **D,** Application of adenosine increased the CV of the NMDA EPSCs (n = 9, p = 0.011, paired t-test). Upper panel shows 10 successive NMDA EPSCs recorded before (*left*) and during (*right*) the application of adenosine. Lower panel shows the calculated CVs from 9 cells (*open circles*) and their averages (*solid circles*). **E,** Intracellular application of GDP-β-S via the recording pipettes did not significantly alter adenosine-induced depression of AMPA EPSCs (n = 6, p<0.001 vs. baseline, paired t-test).

We next tested whether adenosine-mediated depression of AMPA EPSCs is due to inhibition of presynaptic glutamate release or the function of postsynaptic AMPA receptors by performing the following experiments. First, we measured the coefficient of variation (CV) of the AMPA EPSCs before and during the application of adenosine because changes in presynaptic transmitter release are usually concomitant with alterations in CV [48], [49]. CV was significantly increased during the application of adenosine (control: 0.098±0.009, adenosine: 0.160±0.014, n = 7, p = 0.003, Fig. 3A). Second, we measured the PPR of AMPA EPSCs before and during the application of adenosine because a change in presynaptic release probability usually accompanies with an alteration of PPR [50]. Application of adenosine significantly increased the PPR (control: 0.478±0.080, adenosine: 0.633±0.079, n = 10, p<0.001, Fig. 3B). Third, we recorded NMDA EPSCs on the basis that if adenosine inhibited AMPA EPSCs via a presynaptic mechanism, it should also reduce NMDA EPSCs. Bath application of adenosine (100 µM) decreased the amplitudes of NMDA EPSCs (30±4% of control, n = 9, p<0.001, Fig. 3C). The CV of the NMDA EPSCs was also significantly increased during the application of adenosine (control: 0.059±0.013, adenosine: 0.137±0.026, n = 9, p = 0.011, Fig. 3D). These data together indicate that adenosine-mediated depression of AMPA EPSCs is mediated by a reduction of presynaptic glutamate release.

Our results showed that adenosine inhibits presynaptic glutamate release via activation of A_1_ ARs. We next tested whether the involved A_1_ ARs are located presynaptically or postsynaptically because it is possible that adenosine activates postsynaptic A_1_ ARs to generate retrograde messenger(s) to inhibit presynaptic glutamate release. If so, postsynaptic application of the G protein inactivator, GDP-β-S, via the recording pipettes should inhibit postsynaptic A_1_ ARs and block adenosine-induced depression because A_1_ ARs are G protein-coupled. We included GDP-β-S (4 mM) in the recording pipettes and waited for >20 min after the formation of whole-cell configuration to permit the dialysis of GDP-β-S. Under these circumstances, bath application of adenosine (100 µM) still inhibited AMPA EPSCs (30±6% of control, n = 6, p<0.001, Fig. 3E) suggesting that it is unlikely that a postsynaptic retrograde messenger is involved.

### Adenosine inhibits mEPSCs

**Figure 4:**
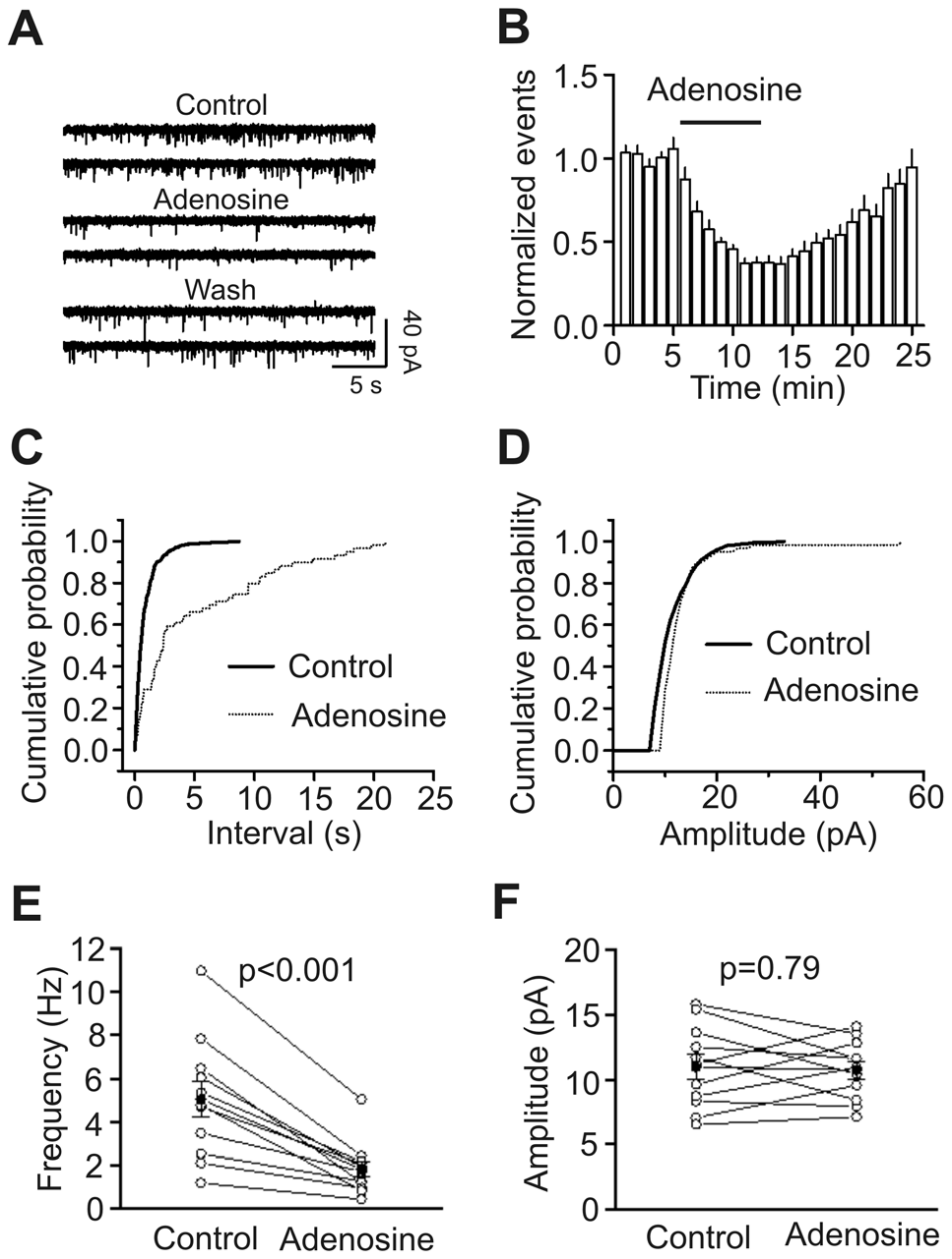
Adenosine decreases mEPSC frequency with no effects on mEPSC amplitudes. A, mEPSCs recorded from a layer III pyramidal neuron in the presence of TTX (1 µM) before, during and after application of adenosine (100 µM). **B,** Time course of the mEPSC frequency averaged from 11 cells. Numbers of mEPSCs at each min were normalized to that of mEPSCs in the 5 min prior to the application of adenosine (n = 11, p<0.001 vs. baseline, paired t-test). **C,** Cumulative frequency distribution from a layer III pyramidal neuron before (*solid*) and during (*dotted*) the application of adenosine. Note that adenosine increased the intervals of the mEPSC (decreased mEPSC frequency, p<0.001, Kolmogorov-Smirnov test). **D,** Cumulative amplitude distribution from the same cell before (*solid*) and during (*dotted*) the application of adenosine (p = 0.08, Kolmogorov-Smirnov test). **E,** Summarized data for adenosine-induced reduction of mEPSC frequency (n = 11, paired t-test). **F,** Adenosine failed to alter significantly mEPSC amplitudes (n = 11, paired t-test).

Adenosine-induced depression of glutamate release could involve action potential-dependent and/or action potential-independent processes. We then tested the effects of adenosine on mEPSCs recorded in the presence of TTX (1 µM) because mEPSCs are action potential-independent. Bath application of adenosine (100 µM) significantly inhibited mEPSC frequency (37±3% of control, n = 11, p<0.001, Fig. 4A, 4B, 4C, 4E). KS test demonstrated that the frequency cumulative probability for each of the 11 cells was significantly inhibited. Whereas 4 cells displayed significant inhibition on the amplitude cumulative probability, pooled data demonstrated that adenosine failed to significantly alter mEPSC amplitude (102±7% of control, n = 11, p = 0.79, Fig. 4D, 4F). Because mEPSC amplitude represents quantal size, these results also suggest that adenosine does not change the quantal size. The results that adenosine inhibited mEPSC frequency suggest that action potential-independent mechanism is also involved in adenosine-induced inhibition of glutamatergic transmission in the EC although the mechanism underlying adenosine-induced depression of glutamate release at other synapses is generally considered to be action potential or Ca^2+^-dependent [20], [51], [52], [53].

### Adenosine inhibits the number of readily releasable vesicles and release probability without changing the rate of recovery from vesicle depletion

**Figure 5:**
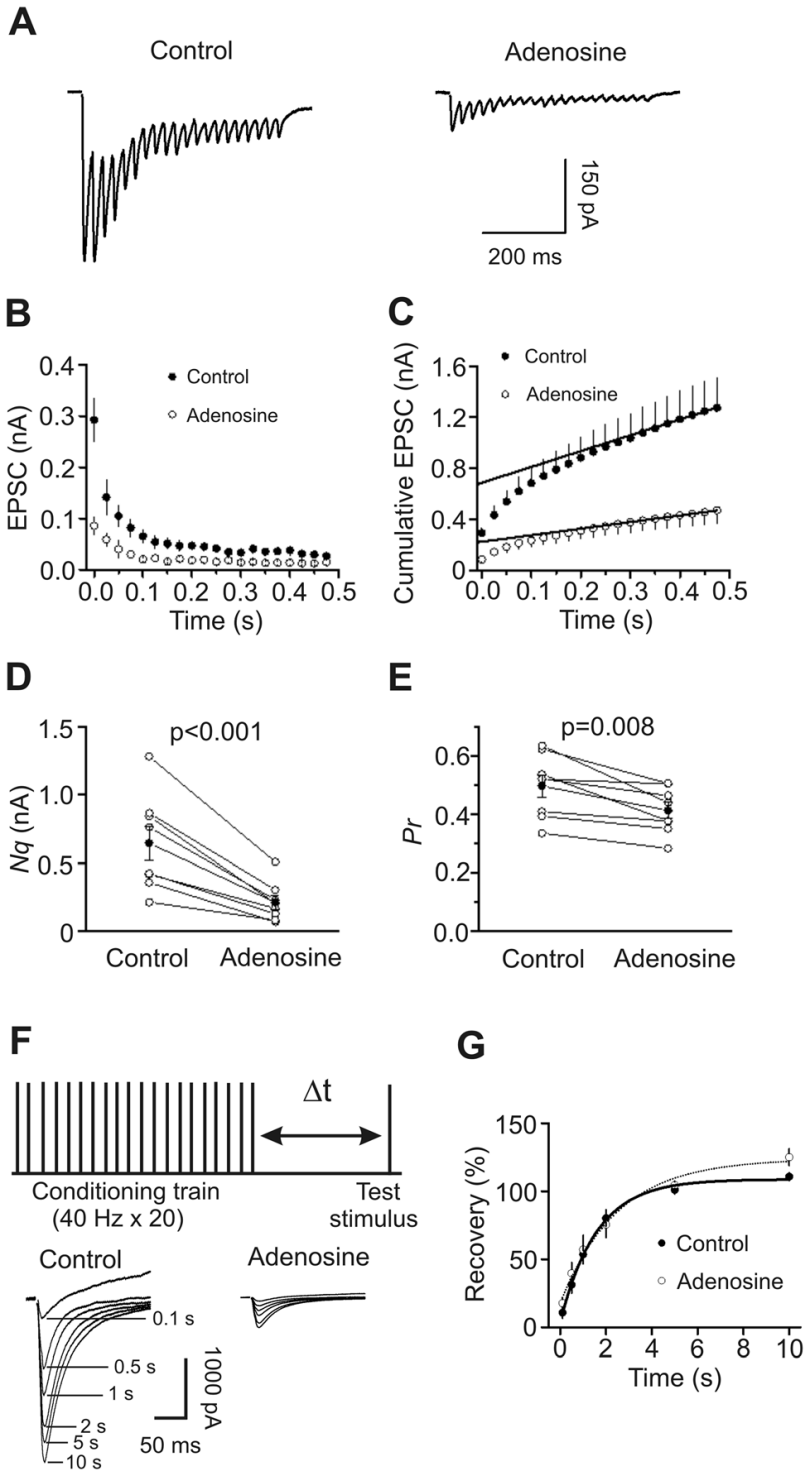
Adenosine decreases the number of releasable vesicles and release probability without changing the rate of recovery from vesicle depletion. A, EPSC trains averaged from 10 traces evoked by 20 stimuli at 40 Hz before (*left*) and during (*right*) the application of adenosine. Stimulation artifacts were blanked for clarity. **B,** EPSC amplitudes averaged from 8 cells in response to 20 stimuli at 40 Hz before and during the application of adenosine. The amplitude of EPSC evoked by each stimulus was measured by resetting the base line each time at a point within 0.5 ms before the beginning of each stimulation artifact. **C,** Cumulative amplitude histogram of EPSCs. For each cell, the last 6 EPSC amplitudes were fit with a linear regression line and extrapolated to time 0 to estimate the readily releasable pool size (*Nq*). **D,** Adenosine decreases *Nq* (n = 8, paired t-test). **E,** Adenosine decreases release probability (*P_r_*, n = 8, paired t-test). For each cell, *P_r_* was calculated as the ratio of the first EPSC amplitude divided by its *Nq* obtained by linear fitting of the cumulative EPSC histogram. **F,** *Upper:* experimental protocol. A conditioning train (20 stimuli at 40 Hz) was followed by a test stimulus. The intervals between the end of the conditioning train and the beginning of the test stimulus were 0.1 s, 0.5 s, 1 s, 2 s, 5 s or 10 s. The interval between each sweep containing the conditioning train and the test stimulus was 30 s to allow the refilling of the synaptic vesicles. *Lower:* EPSCs evoked by the test pulse from the same synapse at different intervals were aligned and superimposed before (*left*) and during (*right*) application of adenosine. Stimulation artifacts were blanked and labels for the traces in the presence of adenosine were omitted for clarity. **G,** Time course of recovery from depletion before and during the application of adenosine expressed as percentage recovery = (I_test_−I_ss_)/(I_1st_−I_ss_)×100, where I_test_ is the EPSC evoked by the test pulse, I_ss_ is the steady-state current left after the conditioning train (the average of the last 5 EPSC evoked by the conditioning train), I_1st_ is the EPSC evoked by the 1^st^ stimulus of the conditioning train. Data before (*thick line*) and during (*thin line*) the application of adenosine from 6 cells were fit by a single exponential function.

Decreases in presynaptic transmitter release can result from a decrease in the number of readily releasable quanta (synaptic vesicles) (*N*) or a decrease in release probability (*P_r_*). We next used the method of high-frequency stimulation [54], [55] to evaluate adenosine-induced changes in *N* and *P_r_*. This method is based on the assumption that high-frequency stimulation-induced depression is primarily caused by the depletion of readily releasable quanta which could be estimated by calculating the cumulative EPSC amplitude for time intervals that are short with respect to the time required for recovery from depression. The zero time intercept of a line fitted to a cumulative amplitude plot of EPSCs equals to the product of *N* and the quantal size (*q*). *P_r_* can be estimated from the first EPSC amplitude divided by *Nq*. Figure 5A shows the EPSC trains evoked by 20 stimuli at 40 Hz before and during the application of adenosine. The average data from 8 cells for the 20 stimuli are shown in Figure 5B. Figure 5C shows the cumulative amplitude histogram. Adenosine decreased *Nq* by 68±3% (n = 8, p<0.001, Fig. 5D) and *P_r_* by 16±4% (n = 8, p = 0.008, Fig. 5E). Because adenosine did not change quantal size (*q*) (Fig. 4D and 4F), these results suggest that adenosine decreases both the number of readily releasable quanta (*N*) and release probability (*P_r_*).

Decreases in the number of readily releasable quanta can occur with or without a concomitant decrease in the rate of vesicle replenishment. We next tested whether adenosine decreases the rate of recovery from vesicle depletion. We employed a protocol comprising a train of stimulation (40 Hz, 20 stimuli) to deplete the readily releasable pool followed by a test pulse at various intervals (0.1 s, 0.5 s, 1 s, 2 s, 5 s, 10 s) to evaluate the replenishment of synaptic vesicles from depletion (Fig. 5F). The time course of recovery after the 40 Hz train could be fitted by a single exponential function with a time constant of 1.7±0.2 s before and 2.9±1.2 s during the application of adenosine (n = 6, p = 0.32, Fig. 5G) indicating that adenosine does not decrease the rate of recovery from vesicle depletion.

### Signaling mechanisms

**Figure 6:**
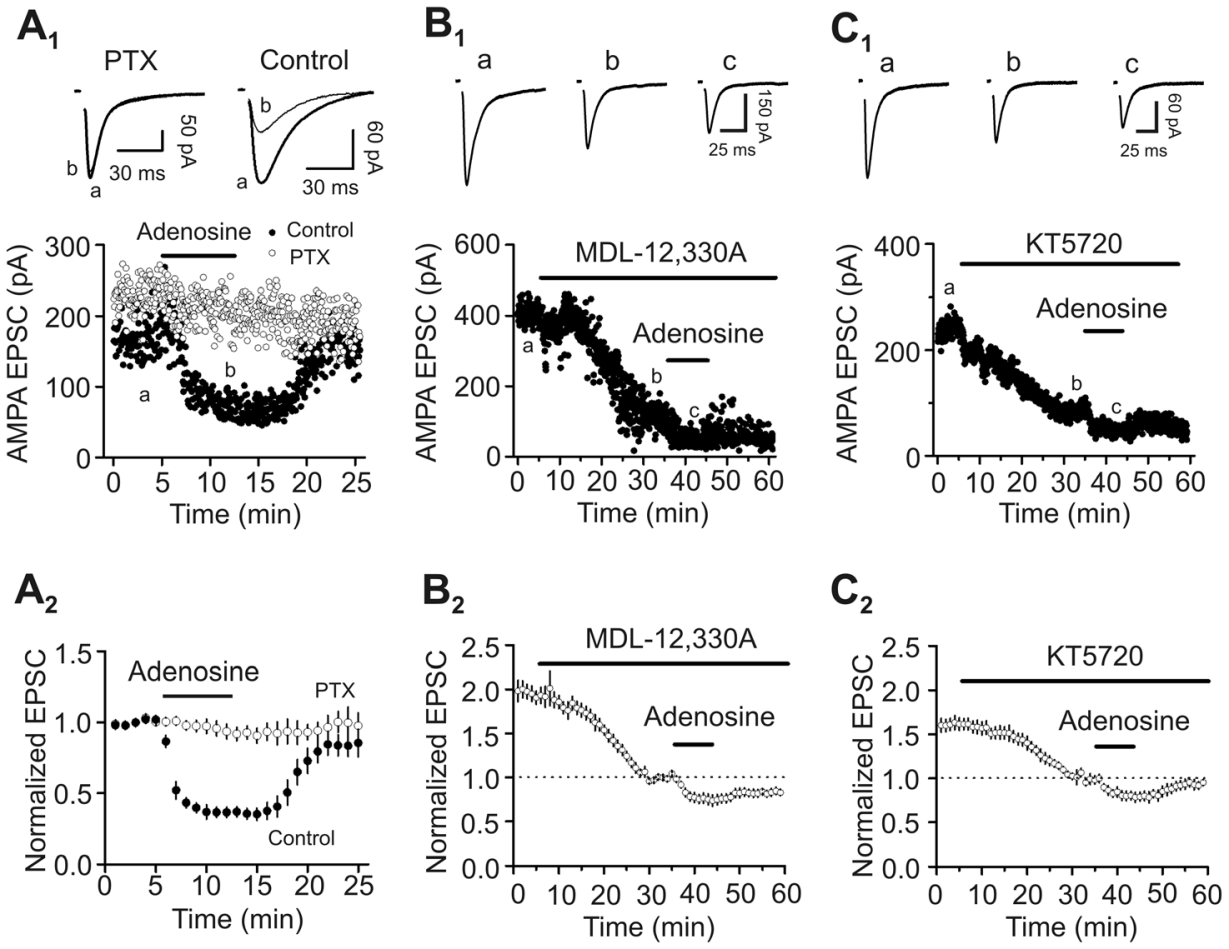
Roles of AC-cAMP-PKA pathway in adenosine-induced depression of glutamate release. A_1_–A_2_, Application of adenosine did not inhibit AMPA EPSCs in slices pretreated with PTX but still induced robust inhibition of AMPA EPSCs in slices undergone the same fashion of treatment without PTX. **A_1_,** AMPA EPSC amplitudes recorded every 3 s before, during and after the application of adenosine. Slices were pretreated with PTX in the extracellular solution bubbled with 95% O_2_ and 5% CO_2_ for ∼10 h (*empty circles*). For control (*solid circles*), slices underwent the similar treatment without PTX. Upper panel shows the average of 10 EPSCs at different time points in the figure. **A_2_,** Averaged data. **B_1_–B_2_,** Bath application of MDL-12,330A (50 µM) alone significantly reduced AMPA EPSCs and reduced adenosine-induced depression of AMPA EPSCs. **B_1_,** Raw data from one cell. **B_2_,** Averaged data from 5 cells. **C_1_–C_2_,** Bath application of KT5720 (1 µM) alone significantly reduced AMPA EPSCs and reduced adenosine-induced depression of AMPA EPSCs. **C_1_,** Raw data from one cell. **C_2_,** Data averaged from 5 cells.

Because A_1_ ARs are coupled to Gα_i_ proteins [20], [21], we tested whether the function of Gα_i_ proteins is required for adenosine-induced depression of glutamate release by applying the Gα_i/o_ inhibitor, pertussis toxin (PTX). Slices were pretreated with PTX (5 µg/ml) for ∼10 h and application of adenosine (100 µM) to the pretreated slices failed to decrease AMPA EPSCs (93±5% of control, n = 7, p = 0.26, Fig. 6A_1_–A_2_). However, application of adenosine (100 µM) to the slices undergone the same fashion of treatment without PTX still inhibited AMPA EPSCs (36±4% of control, n = 6, p = 0.001, Fig. 6A_1_–A_2_). These data together indicate that Gα_i_ proteins are required for adenosine-induced depression of glutamate release.

Activation of Gα_i_ proteins mediated by A_1_ ARs results in depression of AC and subsequent inhibition of PKA [20], [21]. We next tested whether AC and PKA are involved in adenosine-induced depression of glutamate release. Bath application of the AC inhibitor, MDL-12,330A (50 µM) for 30 min significantly reduced AMPA EPSCs (55±2% of control, n = 5, p<0.0001, Fig. 6B_1_–B_2_). Following the inhibition induced by MDL-12,330A, application of adenosine induced a smaller scale of depression (74±6% of control, n = 5, p<0.0001 vs. control without prior application of MDL-12,330A, 37±2% of control, n = 15) suggesting that AC contributes significantly to adenosine-induced suppression of glutamate release (Fig. 6B_1_–B_2_). Furthermore, bath application of the selective PKA inhibitor, KT5720 (1 µM) for 30 min also significantly decreased AMPA EPSCs (62±5% of control, n = 5, p = 0.002, Fig. 6C_1_–C_2_) and subsequent application of adenosine further depressed AMPA EPSCs to 78±6% of control (n = 5, Fig. 6C_1_–C_2_) which was significantly smaller than the inhibition induced by adenosine without KT5720 (37±2% of control, n = 15, p<0.0001). These data suggest that PKA also significantly contributes to adenosine-induced inhibition of glutamate release in the EC.

### Adenosine depresses seizure activity induced by picrotoxin in EC slices via PKA pathway

**Figure 7:**
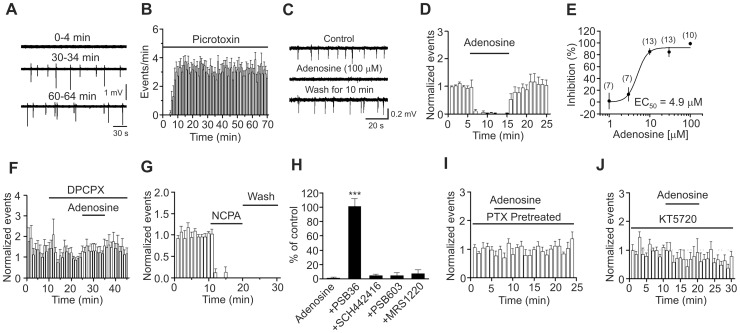
Adenosine-induced depression of seizure activity is mediated by activation of A_1_ ARs and requires the functions of Gα_i_ proteins and PKA. **A,** Seizure events induced by bath application of picrotoxin at the saturated concentration (100 µM) in a rat slice at different times. An extracellular electrode containing ACSF was placed in layer III of the EC to record the seizure events. **B,** Time course of picrotoxin-induced seizure events (n = 7 slices). **C,** Seizure events recorded before, during and after the application of adenosine (100 µM). **D,** Summarized time course of adenosine-induced inhibition of seizure activity (n = 10 slices, p<0.001 vs. baseline, paired t-test). **E,** Concentration-response curve of adenosine-induced depression of seizure activity. Numbers in the parenthesis are the number of slices recorded from. **F,** Prior bath application of the A_1_ AR inhibitor, DPCPX, blocked adenosine-induced depression of seizure events (n = 12 slices, p = 0.89 vs. baseline, paired t-test). **G,** Bath application of the A_1_ AR agonist, NCPA, irreversibly suppressed the seizure events (n = 6 slices, p<0.001 vs. baseline, paired t-test). **H,** Application of antagonists to other ARs except A_1_ ARs did not block adenosine-induced depression of epileptiform activity (One-way ANOVA followed by Dunnett test, *** p<0.001 vs. adenosine alone). **I,** Bath application of adenosine failed to depress significantly picrotoxin-induced seizure events in slices pretreated with PTX (n = 8 slices, p = 0.45 vs. baseline, paired t-test). **J,** Pretreatment of slices with and continuous bath application of the membrane permeable PKA inhibitor, KT5720, blocked adenosine-induced depression of seizure events (n = 8 slices, p = 0.7 vs. baseline, paired t-test).

Adenosine-induced depression of glutamate release could contribute to its antiepileptic effects in the EC. We tested this possibility by using the picrotoxin-induced seizure model in entorhinal slices. We first tested whether picrotoxin-induced seizure events could sustain long-time recording. As shown in Fig. 7A and 7B, seizure events appeared in ∼5 min after the commencement of application of picrotoxin (100 µM) and were stabilized in ∼15–20 min (20 min: 3.1±0.4 events/min, n = 7 slices). Stable seizure activity could be reliably recorded for at least 40 min (at 60 min after the application of picrotoxin: 3.0±0.5 events/min, n = 7 slices, p = 0.85, compared with the events at 20 min after application of picrotoxin, paired t-test, Fig. 7A and 7B). Accordingly, we waited for ∼20 min after the application of picrotoxin to record stable baseline before experiments. Under these circumstances, application of adenosine (100 µM) significantly inhibited the seizure activity (1.3±1.3% of control, n = 10 slices, p<0.001, Fig. 7C and 7D). Because the above experiments were performed on horizontal slices containing the EC, subiculum and hippocampus, one would argue that the inhibitory effect of adenosine on seizure activities recorded from layer III of the EC could be an indirect effect of adenosine on the hippocampus or other brain regions. The following lines of evidence indicate that this is not the case. First, the seizure activity in the horizontal slices containing the above structures originates from the EC [56]. Second, we cut the whole EC out from the horizontal slices under a microscope (denote as ‘mini’ slices) and recorded the seizure activity induced by picrotoxin from layer III of the EC in the mini slices. In this condition, application of adenosine (100 µM) also significantly inhibited the seizure activities (6.3±4.5% n = 12 slices, p<0.001, data not shown) indicating that the inhibitory effect of adenosine originates from the EC. We therefore used the horizontal slices for the rest of experiments simply for the convenience of experiments. The EC_50_ value for adenosine-induced inhibition of seizure activity was measured to be 4.9 µM (Fig. 7E). The inhibitory effect of adenosine on seizure activity was mediated by A_1_ ARs because application of the selective A_1_ AR blocker, DPCPX (1 µM), completely blocked adenosine-induced inhibition of seizure activity (101±10% of control, n = 12 slices, p = 0.89, Fig. 7F). Similarly, application of PSB36 (1 µM), another A_1_ AR antagonist, blocked adenosine-induced suppression of epileptiform activity (101±11% of control, n = 7, p = 0.91, Fig. 7H). Furthermore, application of the selective A_1_ AR agonist, NCPA (2 µM), completely blocked picrotoxin-induced seizure activity (n = 6 slices, p<0.001, Fig. 7G). The irreversible effect of NCPA could be due to its high affinity for A_1_ ARs. We also tested the roles of other ARs in adenosine-mediated antiepileptic effects. Adenosine-induced depression of epileptiform activity was not significantly changed (p>0.05 vs. adenosine alone, Fig. 7H) in the presence of SCH442416 (A_2A_ antagonist, 1 µM, n = 8), PSB603 (A_2B_ antagonist, 1 µM, n = 8) and MRS1220 (A_3_ antagonist, 10 µM, n = 9) indicating that only A_1_ ARs are involved in adenosine-induced depression of epileptiform activity. We further examined the roles of Gα_i_ proteins and PKA in adenosine-induced depression of seizure activity. Application of adenosine (100 µM) did not significantly alter the seizure activity (81±23% of control, n = 8 slices, p = 0.45, Fig. 7I) in slices pretreated with PTX (5 µg/ml for ∼10 h) whereas adenosine still significantly inhibited seizure activity in slices after the same period of treatment without PTX (2.3±1.7% of control, n = 7 slices, p<0.001, data not shown) indicating that Gα_i_ proteins are required for adenosine-induced depression of seizure activity. Moreover, application of adenosine (100 µM) failed to depress significantly seizure activity (83±42% of control, n = 8 slices, p = 0.7, Fig. 7J) in slices pretreated with KT5720 (1 µM for 20 min) demonstrating that PKA is required for adenosine-induced inhibition of seizure activity.

## Discussion

Whereas the EC is an indispensable structure involved in the generation and propagation of epilepsy and adenosine is an endogenous antiepileptic substance, the cellular and molecular mechanisms of adenosine in modulating neural activity in the EC have not been determined. Here, we have shown that adenosine exerts remarkable inhibition on glutamate release in the EC via activation of A_1_ ARs without effects on GABAergic transmission. AC-cAMP-PKA pathway is related to adenosine-induced inhibition of glutamate release. Adenosine-induced inhibition of presynaptic glutamate release in the EC may be mediated by a direct interaction with the presynaptic release machinery. We further demonstrate that adenosine-induced depression of glutamate release is mediated by reductions of glutamate release probability and the number of readily releasable vesicles. Using picrotoxin-induced slice seizure model, we have further shown that bath application of adenosine exerts powerful antiepileptic effects via activation of A_1_ ARs. The functions of Gα_i_ and AC-cAMP-PKA pathway are required for adenosine-induced depression of epileptiform activity suggesting that adenosine-induced inhibition of glutamate release contributes to its antiepileptic effects in the EC.

Whereas adenosine has been shown to suppress the evoked AMPA EPSCs, the effects of adenosine could be due to the inhibition of presynaptic glutamate release and/or postsynaptic AMPA receptors. Our results demonstrate that adenosine inhibits AMPA EPSCs via depression of presynaptic glutamate release based on the following lines of evidence. First, the CV of AMPA EPSCs was significantly increased by adenosine. Second, application of adenosine increased PPR suggesting that adenosine decreases glutamate release probability. Third, when glutamatergic transmission was assessed by measuring NMDA EPSCs, application of adenosine inhibited NMDA EPSCs and the CV of the NMDA EPSCs was also increased in the presence of adenosine. Fourth, application of the G protein inactivator, GDP-β-S, via the recording pipettes to inhibit postsynaptic A_1_ ARs failed to change AMPA EPSCs significantly suggesting that the involved A_1_ ARs are located presynaptically. Finally, application of adenosine inhibited the frequency not the amplitude of mEPSC recorded in the presence of TTX. Because alteration of mEPSC frequency usually suggests a presynaptic mechanism whereas changes of mEPSC amplitude are suggestive of postsynaptic mechanisms, these results further indicate that adenosine inhibits presynaptic glutamate release without changing postsynaptic AMPA receptor functions.

Adenosine-induced reduction of glutamate release could be action potential-dependent and/or action potential-independent. The evoked EPSCs involve both action potential-dependent and action potential-independent processes whereas mEPSCs engage only the action potential-independent release. Our results that adenosine inhibits mEPSC frequency suggest that an action potential-independent mechanism is involved in adenosine-induced depression of glutamatergic transmission. However, adenosine has been shown to inhibit voltage-gated Ca^2+^ channels via A_1_ [20], [51], A_2_ [52] and A_3_ receptors [53]. In this study, we have not examined the contribution of voltage-gated Ca^2+^ channels in adenosine-mediated depression of glutamate release and epilepsy in the EC. However, adenosine-mediated inhibition of voltage-gated Ca^2+^ channels could still be a mechanism. Because our results demonstrate that A_1_ receptors are responsible for adenosine-induced inhibition of glutamate release and epileptic activity, it is reasonable to postulate that if adenosine exerts inhibition on voltage-gated Ca^2+^ channels, it should be mediated via A_1_ receptors as well.

The EC_50_ values underlying adenosine-induced depression of glutamate release and epileptic activity are 3.8 µM (Fig. 2E) and 4.9 µM (Fig. 7E), respectively. The extracellular concentration of adenosine under resting conditions has been estimated to be 1–2 µM in rat and human hippocampi [57]. This concentration is close to the measured EC_50_ values. In this study, we tried to probe the effects of endogenously released adenosine on glutamate release in the EC. Application of DPCPX, the selective A_1_ AR antagonist, alone failed to significantly increases AMPA EPSCs. However, bath application of the adenosine transporter blocker, dipyridamole, significantly reduced AMPA EPSCs and prior application of the selective A_1_ AR antagonist, DPCPX, blocked dipyridamole-induced depression of AMPA EPSCs. These results together suggest that endogenously released adenosine in basal conditions has the potential to inhibit glutamate release although it is quickly removed from the synapses by adenosine transporters. Adenosine levels rise approximately 30-fold higher (65 µM) than basal levels in the human epileptic hippocampus following seizure onset and remain elevated postictally [57]. This concentration of adenosine should exert the maximal antiepileptic effect according to our concentration-response relationship (Fig. 7E). Our results therefore demonstrate that adenosine is an endogenous antiepileptic substance in the EC.

A_1_ ARs are coupled to Gα_i_ proteins resulting in inhibition of AC-cAMP-PKA pathway [20], [21]. Our results demonstrate that this intracellular pathway is involved in adenosine-induced inhibition of glutamate release. Because the effects of adenosine on glutamate release in the EC may include both action potential-dependent and independent mechanisms and the action potential-dependent mechanism involves direct G-protein coupling to voltage-gated Ca^2+^ channels without the requirement of the AC-cAMP-PKA pathway, it is reasonable to postulate that the target of the AC-cAMP-PKA pathway is the release machinery in the EC. Consistent with our results, AC-cAMP-PKA pathway has been shown to enhance exocytosis processes via a direct action on the secretory machinery in a variety of secretory cells [58], [59], [60], [61].

Adenosine has been shown to modulate GABAergic transmission in a variety of neurons including the hypothalamic neurons [62], [63], hippocampal CA1 neurons [64] and tuberomammillary nucleus neurons [65]. However, our results have shown that application of adenosine does not modulate GABAergic transmission onto layer III pyramidal neurons in the EC. Consistent with this result, we have further shown that application of adenosine still exerts robust inhibition on the epileptiform activity induced by the GABA_A_ receptor blocker picrotoxin suggesting that adenosine-mediated antiepileptic effects are mediated by its inhibition on glutamatergic transmission not by its interaction with GABAergic transmission if there is any.

Whereas adenosine has been shown to inhibit epilepsy in several in vivo animal models in the EC via activation of A_1_ ARs [33], [34], the cellular and molecular mechanisms whereby adenosine depresses epilepsy have not been determined. Using the picrotoxin-induced seizure model in the EC slices, we demonstrate that A_1_ ARs, Gα_i_ proteins and PKA are required for adenosine-mediated depression of epileptiform activity. Because these signaling molecules are involved in adenosine-mediated depression of glutamate release, these results suggest that adenosine-induced depression of glutamate release should at least contribute to its antiepileptic effect in the EC. However, this conclusion is based on the data collected from 12- to 18-day-old rats. We chose this age of the animals because it is difficult to induce epileptiform activity in slices cut from rats older than 18 days. We cannot exclude the possibility that the antiepileptic mechanisms of adenosine in adult animals may be different from those found in juvenile animals. Moreover, here we focused on adenosine-mediated inhibition of glutamate release. It is possible that adenosine may have other effects in the EC such as modulating the excitability of entorhinal neurons. Further studies are still required for a comprehensive understanding of the cellular and molecular mechanism underlying adenosine-induced inhibition of epilepsy.
